# COVID-19 Evidence Reviews website: a VA effort to catalog and curate COVID-19 evidence reviews

**DOI:** 10.5195/jmla.2022.1237

**Published:** 2022-01-01

**Authors:** Kathryn Vela

**Affiliations:** 1 kathryn.vela31@gmail.com, Research Health Science Specialist, Evidence Synthesis Coordinating Center, Portland VA Healthcare System, Portland, OR, and Medical Librarian, St. Luke's Health System, Boise, ID

**Keywords:** COVID-19, systematic review, rapid review, Internet-based intervention

## Abstract

**Background:**

The COVID-19 pandemic has sparked a wave of SARS-CoV-2 and COVID-19 research that organizations around the world have been synthesizing in evidence reviews to provide high-quality evidence to support policymakers and clinicians. The urgency of these efforts opens these organizations to the risk of duplicated efforts, which could waste valuable time and resources.

**Case Presentation:**

The VA Evidence Synthesis Program (VA ESP) formed a COVID Response Team that launched an online catalog of COVID-19 evidence reviews in March 2020 (https://www.covid19reviews.org/). The goal of this website is to capture the work of evidence synthesis groups in the US and around the world to maximize their collective contributions to patients, frontline clinicians, researchers, and policymakers during the COVID-19 pandemic and avoid duplicating efforts.

**Conclusions:**

This ongoing and evolving project provides a helpful catalog of evidence reviews at various stages of production; in addition, the website provides further value with informational icons, review collections, and links to similar resources. The VA ESP will maintain this website to continue to support the needs of policymakers, clinicians, and researchers both within the VA and around the world throughout the COVID-19 pandemic.

## BACKGROUND

The beginning of the COVID-19 pandemic in December 2019 launched a wave of research related to this global emergency. As the body of literature grew at an unprecedented rate, the need for evidence syntheses became critical to making sense of the deluge of information. Evidence synthesis groups around the world, including the Cochrane Collaboration [1], McMaster University [2], and UNCOVER [3], began producing systematic and rapid reviews on COVID-19 topics to meet this need.

The VA Evidence Synthesis Program (VA ESP) is another group that faced similar challenges addressing the myriad of information needs health system decision-makers had on the treatment and care for COVID-19 patients. Since 2007, the ESP, a research program embedded in the Department of Veterans Affairs (VA), has focused on improving veteran health and wellbeing through the production of tailored evidence synthesis products to support the implementation of effective practices [4]. The VA ESP in Portland, Oregon, realized early in the pandemic that, given the limited time and resources, cataloging reviews on COVID-19 would allow the program to respond nimbly and effectively to urgent evidentiary needs that arose in the COVID-19 crisis. In March 2020, a multisite, multicenter COVID Response Team was created, and existing relationships with systematic reviewers were leveraged to begin the work of building an online catalog of COVID-19 evidence reviews to capture the work of evidence synthesis groups in the US and around the world. This effort aimed to maximize the collective contributions to patients, frontline clinicians, researchers, and policymakers during the COVID-19 pandemic and avoid duplicating efforts among evidence synthesis groups.

## CASE PRESENTATION

The COVID-19 Evidence Reviews website (https://www.covid19reviews.org/) is updated Monday through Friday (excluding US holidays) by the VA ESP website group. The website group is composed of a medical librarian, three research assistants, and an informatics research associate who contribute approximately ten to fifteen hours per week to maintaining the website. The website is built on Adobe ColdFusion and uses Microsoft SQL to populate the website content. The resources outlined in Table 1 are searched daily or weekly, depending on their update frequency, and eligible evidence reviews are added to the website. The website group sought resources that covered a wide scope, were maintained by high-quality organizations, and provided a consistent way to search for evidence reviews. Given that the website group is embedded within the VA, priority was given to resources and reviews that would be beneficial to the VA COVID-19 treatment and prevention effort. The inclusion criteria for addition to the website include 1) systematic or rapid review, 2) in the protocol, preprint, or publication stage of development, and 3) directly related to a COVID-19 topic.

**Table 1:** Resources used to populate and update the COVID-19 Evidence Reviews website

Resources monitored daily	Resources monitored weekly
NIH COVID-19 Portfolio (MEDLINE, Research Square, medRxiv, SSRN, Preprints.org) prospero	World Health Organization (WHO) Global Literature on Coronavirus Disease database Centre for Evidence-Based Medicine (CEBM) Oxford COVID-19 Evidence Service Cochrane COVID Reviews UNCOVER International Network of Agencies for Health Technology Assessment (INAHTA) member organizations COVID-END

Given that the website includes evidence reviews at multiple stages of production (protocol, preprint, and published), the website group devised a process for ensuring that each evidence review was included on the website only once, at its current stage of production. Potential preprint and published reviews are double-checked in the website database for a previous version; if no existing version is located, then the evidence review is added to the website database. If an existing version were to be located, then that existing version would be updated, rather than creating a new record for the same review. The website group also monitors living reviews for updates to ensure that the links provided on the website connect users to the most recent version of the review. While updates to living reviews may appear in regular searching, a separate list of identified published living reviews is maintained and monitored for updates monthly.

Resources for populating the website are frequently evaluated for relevance to the evidence reviews website. Resources that begin to slow their update frequency or stop updating with new evidence reviews altogether will be removed from the resource monitoring list. Conversely, new resources that meet the needs of the website as previously described will be added to the resource monitoring list. By regularly evaluating the resources on the resource monitoring list, the website group can ensure that the website update process is as efficient as possible.

**Figure 1:**
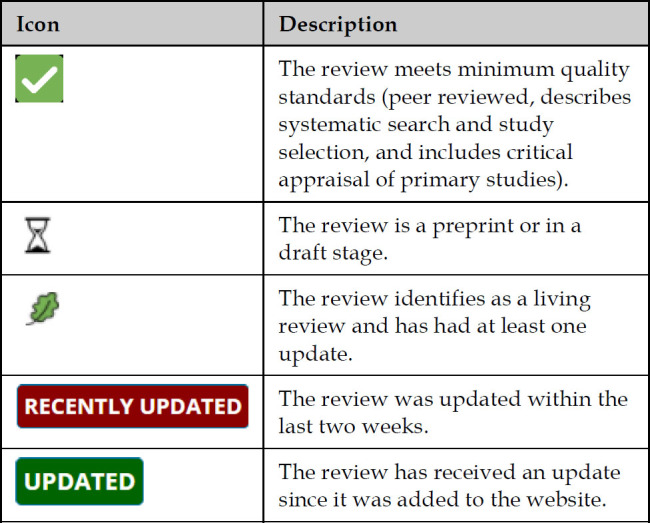
Icons used on the COVID-19 Evidence Reviews website to indicate additional information about evidence reviews.

In addition to providing a catalog of evidence reviews on COVID-19 topics, the website group seeks to add value to these existing reviews by making it easier for users to locate relevant and quality evidence. One way this is accomplished is by employing a small number of icons to provide additional information about reviews, specifically related to quality, updates in status, and whether it is a living review (Figure 1). The website group also curates collections of evidence reviews based on key topics of interest related to the COVID-19 pandemic: Clinical Characteristics, Candidate Therapeutics, Diagnostics, Infection Control, and Mental Health. These collections are readily accessible through a drop-down menu on the homepage of the website and are updated on a weekly basis. New collections are added as needed; for example, the Mental Health collection was added in November 2020 in response to a growing interest and a growth of literature related to mental health outcomes in the COVID-19 pandemic.

Updated features have been added to the website throughout its development in response to feedback from users. An export function was added to allow users to export search results as a CSV file, and a sort function was added that makes it easier for users to view the latest evidence first. The search feature of the website has been improved through multiple iterations of development and now allows users to search for keywords on the entire website or just within a specific section. A “Featured” section was launched to operate as a microblog and draw users' attention to key guideline updates, important resources, and useful website features. The website group also distributes a monthly newsletter to update and remind subscribed users of website features like the collections and to highlight complementary COVID-19 resources.

## DISCUSSION

As the website has been developed, the website group has become aware of and reached out to organizations maintaining similar websites in the spirit of collaboration. The website group currently has partnerships with groups at COVID-END from McMaster University [2] and the COVID-19 Best Evidence Front Door from the University of Michigan [5]. These partnerships involve the exchange of website content to ensure that each resource is as complete and comprehensive as possible. This also helps to prevent the duplication of efforts as partners share information about their scope and purpose. For example, the COVID-END resource has a wide-reaching purpose to support COVID-19 researchers and coordinate synthesis efforts, while the COVID-19 Evidence Reviews website is focused on supporting VA COVID-19 initiatives. The COVID-19 Best Evidence Front Door includes guidelines in its database, which can fill a gap in the COVID-19 Evidence Reviews website without the website group needing to add additional labor to their workflow.

In addition to the resources managed by these partners, there are other similar websites that are monitored and promoted by the website group in the form of a Resources page on the website; this is intended to further support the evidence synthesis efforts of researchers by providing them with easy and accessible links to other evidence synthesis resources. For example, the previously mentioned UNCOVER resource provides a catalog of evidence reviews, as does the Cochrane COVID Reviews website [3,6].

The website has supported the efforts of the VA ESP in providing timely evidence syntheses to VA policymakers several times over the course of the pandemic. As an example, a request was submitted to the VA ESP for a rapid review on the use of ivermectin to treat COVID-19. Before the VA ESP devoted time and resources to this request, the website was searched for existing reviews on the topic. A relevant systematic review was located and provided to the administrator who made the request, and it ultimately met their information needs. The use of the website prior to conducting a new rapid review made it easier for the VA ESP to find a quality review on the requested topic and prevented the creation of a duplicate review.

In a broader sense, the COVID-19 pandemic and accompanying infodemic have magnified the value that librarians can add to health care systems by facilitating access to high quality evidence [7]. With the increase in information, and especially misinformation, resulting from the pandemic, the ability of librarians to identify appropriate resources and locate relevant research has become a critical part of health care systems' ability to make evidence-based decisions [8]. Resources like the VA ESP website and similar websites are examples of how librarians and information professionals can be valuable partners within the health system by providing convenient and timely access to reliable information that is critical to creating responsible health care policies.

As of June 9, 2021, the VA ESP website provided access to 6,800 reviews at the protocol, preprint, and publication stages of development. Over 6,500 users from 111 countries have accessed the website, and the website's monthly newsletter has 80 subscribers. The website group on the VA ESP COVID-19 Response Team will continue to update and improve the website through the COVID-19 pandemic and beyond, to provide a useful evidence synthesis resource to clinicians, policymakers, and researchers around the world.

## Data Availability

There are no data associated with this article.
